# BiPOD arthroscopically assisted bidirectional stabilisation technique for high-grade acromioclavicular joint injury: two-year clinical and radiological outcomes

**DOI:** 10.1007/s00402-021-03768-5

**Published:** 2021-02-08

**Authors:** Richard J. Murphy, Benedikt Ambuehl, Michael O. Schaer, Johannes Weihs, Beat K. Moor, Matthias A. Zumstein

**Affiliations:** 1 Shoulder, Elbow and Orthopaedic Sports Medicine, Orthopaedics Sonnenhof, 3006 Bern, Switzerland; 2 Shoulder, Elbow and Orthopaedic Sports Medicine, Department of Orthopaedic Surgery and Traumatology, Inselspital, Bern University Hospital, University of Bern, 3010 Bern, Switzerland; 3 Stiftung Lindenhof I Campus SLB, Swiss Institute for Translational and Entrepreneurial Medicine, Freiburgstrasse 3, 3010 Bern, Switzerland; 4 Centre Hospitalier du Valais Romand, Hôpital du Valais (RSV), Hôpital de Martigny, av. de la Fusion 27, 1920 Martigny, Switzerland

**Keywords:** Acromioclavicluar joint injury, BiPOD, Shoulder, Rockwood, Surgical technique

## Abstract

**Purpose:**

The purpose of this study was to evaluate the intermediate-term clinical and radiological outcomes for acute, unstable acromioclavicular joint (ACJ) injuries treated with the arthroscopically assisted BiPOD stabilisation technique.

**Methods:**

Twenty-three patients who sustained acute, unstable ACJ injuries were included in this prospective study. We recorded demographics, injury classification, time to surgery, clinical scores, radiological outcomes and complications; each patient completed a minimum of 2 years post-operative observation.

**Results:**

Mean follow-up was 26 months (range, 24—34). Clinical outcomes scores demonstrated good 2-year results: relative Constant score, 97.9/100; ACJ Index, 89.4/100; Subjective Shoulder Value, 92.4/100 and Taft = 11.1/12. Final C–C distance showed a mean of 0.7 mm (SD ± 1.8 mm) at 2 years. Complication rate was 9%.

**Conclusion:**

The BiPOD technique shows excellent, reliable intermediate-term results with a favourable complication rate compared to existing techniques; it provides a comprehensive surgical option for the stabilisation of acute ACJ injuries restoring both vertical and horizontal stability.

## Introduction

Complete dislocation of the acromioclavicular joint (ACJ) is common, with an incidence of 3–4 per 100,000; around 50% of these dislocations occur during sporting activities, commonly in overhead and contact sports [1]. They are most commonly classified using the Rockwood system [2]. The clavicle is stabilised by the ACJ capsular ligaments, predominantly in the horizontal (axial) plane, the trapezoid and conoid coracoclavicular ligaments also confer horizontal stability, although their main stabilising action is in a vertical (coronal) plane; during injury, the ACJ becomes more unstable as each additional ligament ruptures [3]. There remains controversy over the best course of treatment for these injuries, particularly for the moderately displaced Rockwood III injuries, although international consensus on a pragmatic pathway for assessment and management was published by ISAKOS in 2014 [4].

Once a decision has been taken to proceed with surgical stabilisation of an acute, unstable ACJ injury, the next question to answer is the form that stabilisation should take. Myriad techniques for ACJ stabilisation have been suggested and tested including Bosworth screw fixation of the clavicle to the coracoid [5], suture sling reconstruction of the coracoclavicular ligaments [6], muscle transfer [7], coracoacromial ligament transfer [8], tendon graft [9], hook plate [10], trans-coracoid tightrope fixation[11–13] and synthetic ligament sling reconstruction [14], to mention only a few of the published methods. Only one of these techniques, other than perhaps hook plate fixation, address both the horizontal and vertical instability of the injury caused by ACJ capsular ligament and coracoclavicular ligament disruption respectively[13]; the focus of most techniques remains on stabilising only in a vertical plane. As such, it is not surprising that persisting horizontal instability is seen post-operatively, in addition to recurrent vertical instability [12].

**Fig. 1:**
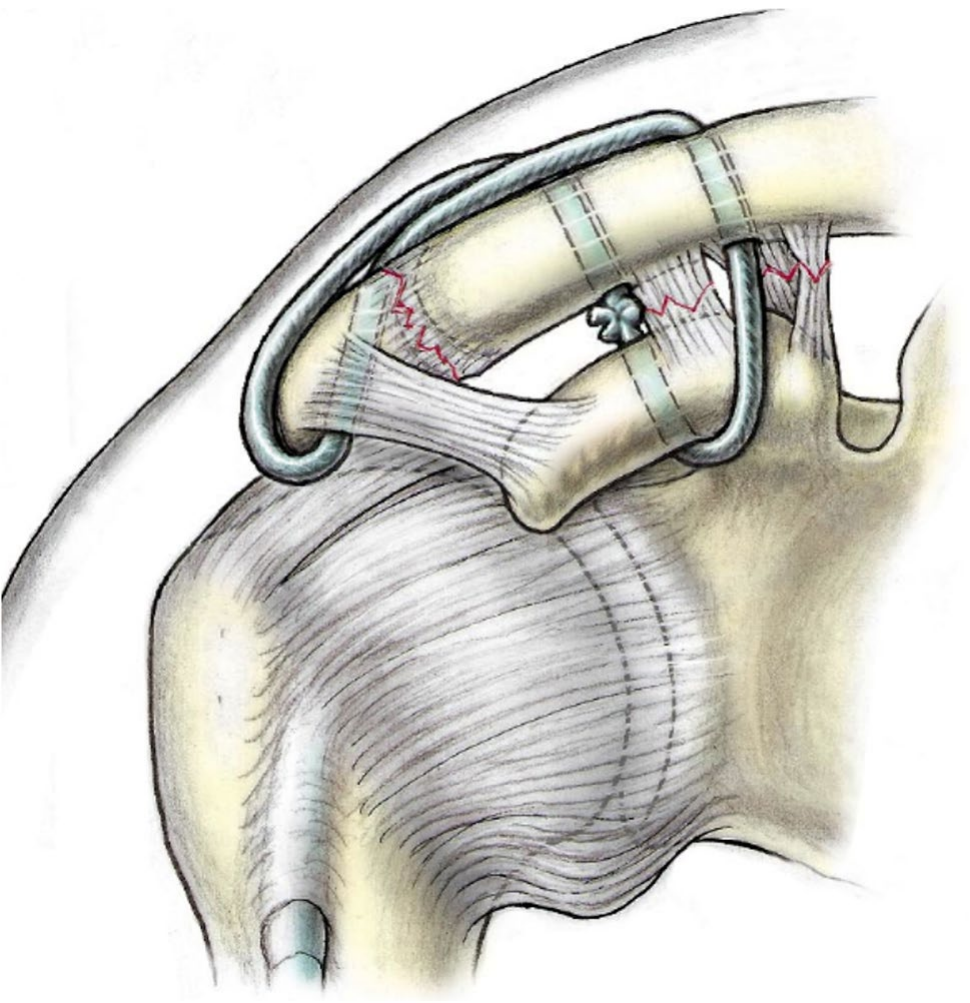
BiPOD acromioclavicular joint stabilisation technique. Figure-of-eight suture construct creates a sling beneath the coracoid with both limbs passing through the clavicle to reconstruct the coracoclavicular ligaments and passes through and around the acromion to reconstruct the acromioclavicular joint capsular ligaments. Knots can be tied beneath (as shown) or above the clavicle

The BiPOD technique is an arthroscopically assisted method for stabilisation and reconstruction of both the coracoclavicular and acromioclavicular capsular ligaments [15], similarly to Scheibel’s recently reported technique [13]. The method employs a suture tape figure-of-eight construct creating a sling beneath the coracoid, passing through two vertical clavicular tunnels and laterally through and around the acromion, thus reconstructing both the coracoclavicular and acromioclavicular capsular ligaments and restoring both vertical and horizontal stability, see Fig. 1. In biomechanical testing, the BiPOD technique showed similar performance to double tightrope methods [16].

We have employed this technique in our institution as our surgical treatment for unstable acute ACJ injuries for a number of years and this study aimed to present the intermediate term clinical and radiological outcomes of a cohort of patients having undergone BiPOD ACJ stabilisation.

## Materials and methods

### Study protocol

This prospective observational case series was undertaken in compliance with local regulations and received ethical approval (Approval number KEK 154/14).

We included patients presenting to our institution with acute high-grade unstable ACJ injuries requiring surgical management as indicated by the ISAKOS guidelines [4]; unstable Rockwood IIIb injuries, plus all Rockwood IV injuries or greater were offered surgical stabilisation. We included participants of at least 18 years of age who were operated upon within 3 weeks of injury; we excluded those who were polytraumatised, not suitable for surgery due to their comorbidities, pregnant or unable to give informed consent.

Twenty-seven patients underwent BiPOD ACJ stabilisation surgery during the study period; four were followed up elsewhere and could not be included in the analysis due to lack of data. The remaining 23 patients contributed to the study cohort and were followed up for a minimum of 2 years. Each patient underwent acute ACJ stabilisation surgery, performed by one of the two senior authors (MAZ and BKM) using the BiPOD technique [15]. We recorded demographic details, injury classification, time to surgery, clinical scores, radiological outcomes and complications observed. Postoperative clinical assessments were performed by the two senior authors (MAZ and BKM).

### Operative technique

The arthroscopically assisted BiPOD technique has been previously described in detail [15]. The technique involves reconstruction of both the coracoclavicular and acromioclavicular capsular ligaments using a multi suture construct arranged in a figure-of-eight pattern as show in Fig. 1; we use two FibreTape sutures (Arthrex, Naples, Florida) and a Poly-Tape (Neoligaments, Leeds, UK). The suture material passes through two vertical drill holes in the clavicle and beneath the coracoid as a sling, with arthroscopic assistance. The free ends of the FibreTape and Poly-Tape sutures are tied following debridement, mobilisation and manual reduction of the ACJ, this reconstructs the coracoclavicular ligaments. The knots can be tied beneath the clavicle with a small open approach or above the clavicle with a minimally invasive approach. From an open incision superior to the clavicle, one end of both tapes is then passed through a drill hole in the acromion to the subacromial space, retrieved through a lateral arthroscopy portal and advanced subcutaneously around the lateral border of the acromion to return to the clavicle; the free ends are tied once again, reconstructing the acromioclavicular capsular ligaments.

Postoperatively, patients were immobilised, adducted in a sling for 6 weeks with early physiotherapy to include passive and active assisted range of motion exercises; flexion and abduction were limited to 60° until 3 weeks and gradually increased to 90° by 6 weeks. Activities that apply strain to the ACJ were avoided until 12 weeks, as was heavy lifting.

### Radiographic outcomes

**Fig. 2:**
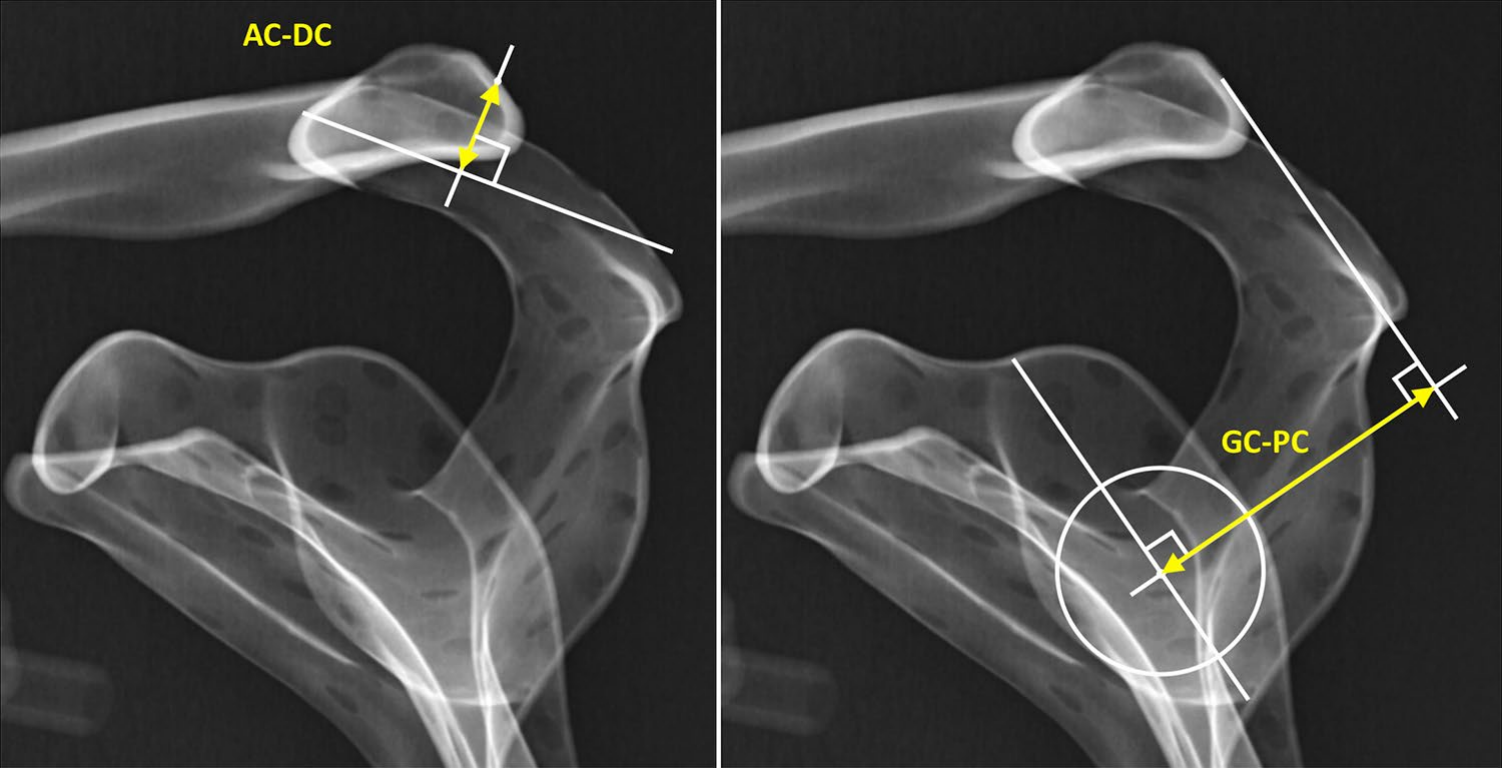
Illustration of acromial centre line to dorsal clavicle (AC-DC) and glenoid centre line to posterior clavicle (GC-PC) measurements [19]

Radiographs included a panoramic Zanca anteroposterior view [17] at the time of injury and 3, 6, 12 and 24 months post-operatively to evaluate coracoclavicular distance [2] and bilateral Alexander views [18] (lateral stress radiographs) at the time of injury and at 24 months post-operatively to evaluate acromial centre line to dorsal clavicle (AC-DC) and glenoid centre line to posterior clavicle (GC-PC) as measures of vertical and horizontal ACJ displacement respectively [19]. Figure 2 shows how the AC-DC and GC-PC measurements are taken. The AC-DC and GC-PC measurements have been shown to be reliable up to a radiographic projectional error of 20° in each anatomical plane, making this a highly accurate and reproducible means of evaluating both horizontal and vertical displacement of the ACJ [19]. Measurements and classifications were made by two members of the research team (BA and JW) and recorded as the difference between injured and uninjured sides.

### Clinical outcomes

All patients completed a number of clinical outcome scores at 3, 6, 12 and 24 months post-operatively. We deemed scores performed at the time of injury to be of limited value. Scores included relative Constant score [20], acromioclavicular joint index [12], subjective shoulder value (SSV) [21] and the Taft score [22].

### Statistical analysis and data management

Data recording and storage met with local guidelines for data security and confidentiality.

We recorded all individual radiographic measurements to 0.1 mm and present results as means with standard deviation (SD) or 95% confidence interval (95% CI) as indicated. We assessed normality using a Kolmogorov-Smirnoff test prior to parametric testing and used non-parametric tests as indicated.

Radiographic measurement groups all passed normality testing; we compared pre- and post-operative radiographic measurements using unpaired t-tests to allow for any missing data points and applied a Welch correction [23] to accommodate variation in standard deviation between the comparator groups.

Clinical scores showed varying degrees of normality so were analysed using a Kruskal–Wallis test with 6-, 12- and 24-month scores compared against 3-month scores as a baseline; a Dunn correction was applied to account for the multiple intergroup comparisons.

We performed statistical analyses using “R: a language and environment for statistical Computing” (R Core Team, 2016. R Foundation for Statistical Computing, Vienna, Austria) and “GraphPad Prism version 8 for mac OS” (GraphPad Software, La Jolla California USA, www.graphpad.com).

## Results

### Demographics, injury and treatment

Twenty-three patients were included in the analysis; 20 were male and 3 were female, with a mean age of 41 years at the time of injury (range 21–76 years) and a mean follow-up of 26 months (range 24–34 months). Thirteen injuries were left-sided and 10 right-sided and classified as Rockwood IIIb: 3 cases, Rockwood IV: 2 cases and Rockwood V: 18 cases. Time to surgery post injury showed a mean of 9 days (range 5–18 days). All patients underwent acute BiPOD ACJ stabilisation as described previously, without significant intra-operative complications, other than a single suture rupture during manipulation, which was immediately replaced.

### Clinical outcomes

**Fig. 3:**
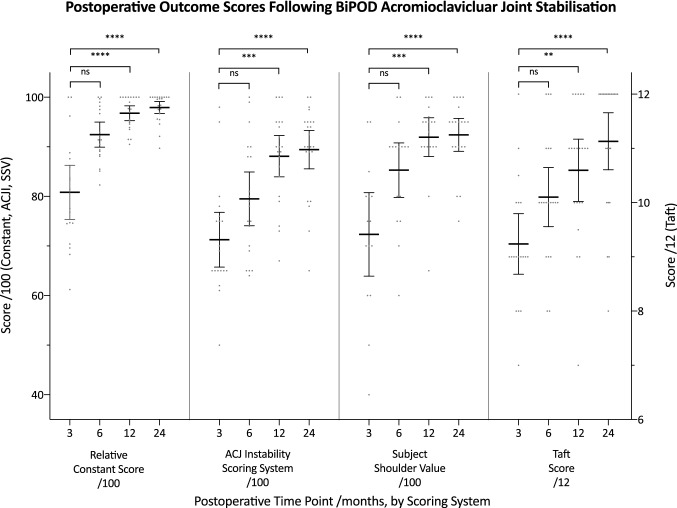
Physician- and patient-reported outcome scores following BiPOD ACJ stabilisation. All scores show statistically significant and maintained improvement compared to baseline (3 months post-operatively) from 12 months onwards (*ACJI* acromioclavicular joint index, *SSV* subjective shoulder value)

All outcomes measures, physician and patient reported, showed improvement post-operatively from 3 months to 2 years with statistically significant improvements seen from 12 months onwards for all scores compared with 3 months post-operatively as a baseline, see Fig. 3. From 3 to 24 months post-operatively, mean relative Constant score improved by 17.1 points from 80.8 (SD ± 11.0) to 97.9 (SD ± 2.8), mean ACJ index improved by 18.1 points from 71.3 (SD ± 11.4) to 89.4 (SD ± 8.9), mean Subjective Shoulder Value improved by 20.1 points from 72.3 (SD ± 15.2) to 92.4 (SD ± 7.1) and mean Taft score improved by 1.9 points from 9.2 (SD ± 1.2) to 11.1 (SD ± 1.2).

### Radiographic outcomes

**Fig. 4:**
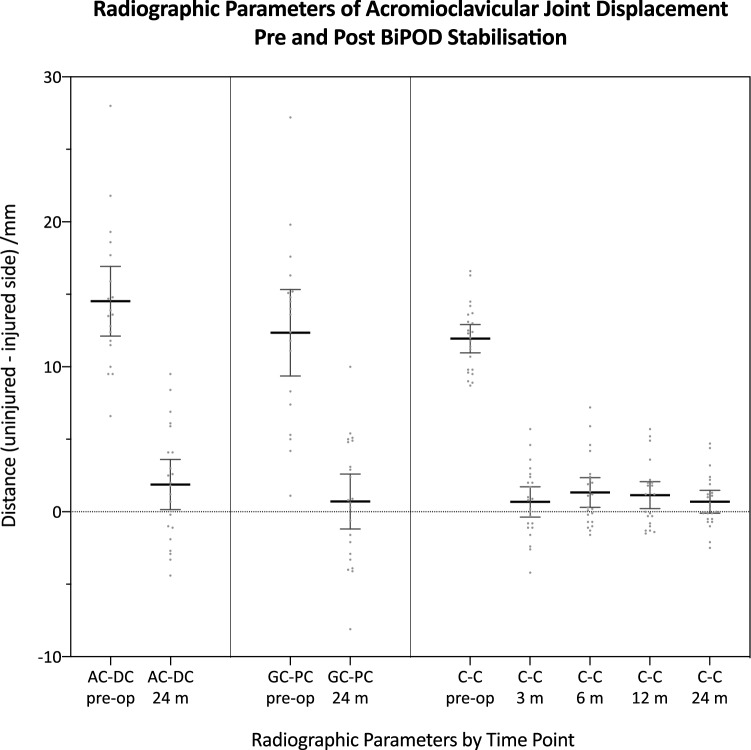
Radiographic parameters of ACJ displacement pre- and post-operatively following BiPOD ACJ stabilisation; all measurements recorded as the difference between injured and uninjured sides. *AC-DC* acromial centre line to dorsal clavicular cortex distance, *GC-PC* glenoid centre line to posterior clavicular cortex distance, *C-C* coracoclavicular distance

All radiographic parameters of ACJ displacement (C-C, AC-DC and GC-PC), measured as difference between injured and uninjured sides, showed significant improvement 2 years post-operatively compared with pre-operative measurement at the time of injury. Measurements of C-C distance showed a consistent degree of restoration of ACJ position at all post-operative time points. AC-DC difference, as a measure of vertical displacement, improved from a pre-operative mean of 14.5 mm (SD ± 5.0 mm) to 1.9 mm (SD ± 3.9 mm) at 2 years. GC-PC difference, as a measure of horizontal displacement, improved from a pre-operative mean of 12.4 mm (SD ± 6.2 mm) to 0.7 mm (SD ± 4.3 mm) at 2 years. C-C distance difference, as a commonly used measure of vertical displacement, improved from a pre-operative mean of 11.9 mm (SD ± 2.3 mm) to 0.7 mm (SD ± 1.8 mm) at 2 years, the maximum C-C distance measured at final follow up was 4.7 mm. There was some degree of rebound displacement seen between 3 and 6 months, although this was small and not statistically significant, with a mean of 0.7 mm (SD ± 1.5 mm), and the C-C distance followed a trend to settle towards zero again between 6 months and 2 years. The radiological outcomes for all cases are summarised in Fig. 4.

### Complications

Two patients were treated for post-operative wound problems. One patient required a short course of oral antibiotics following presentation with erythema and a subsequent positive wound swab. A second patient required surgical debridement and wound closure due to dehiscence in the early post-operative period; this same patient also sustained an ipsilateral clavicle fracture 12 months post BiPOD stabilisation, due to an additional, unrelated traumatic injury, requiring open reduction and internal fixation. Both patients recovered well and had clinical and radiological outcomes in keeping with the remaining cohort.

## Discussion

The arthroscopically assisted BiPOD technique for ACJ stabilisation aims to reconstitute both the coracoclavicular and acromioclavicular capsular ligaments to restore both vertical and horizontal stability respectively to the ACJ following an unstable injury pattern. In this case series, the intermediate term clinical and radiological outcomes following the BiPOD procedure were excellent with minimal complications observed.

Clinical outcomes following BiPOD surgery were shown to be good with both physician and patient reported measures showing extremely high scores at 2 years post-surgery, with significant improvements in scoring throughout the intermediate term postoperative period. Two-year results yielded excellent absolute scores (relative Constant score = 97.9/100, ACJI = 89.4/100, SSV = 92.4/100, Taft = 11.1/12); each score demonstrated a similar pattern of improvement over the course of post-operative monitoring, suggesting that each of these measures offers a reasonable insight into patient recovery from ACJ stabilisation surgery. These results compare favourably with the clinical outcomes following the majority of existing and commonly used alternative surgical techniques [11, 12, 24].

In terms of complications, we saw two wound problems post-operatively from the 23 patients (9%). One of these patients required a return to theatre, although, in cases of routine recovery, this method required only a single operation, unlike many that necessitate metalwork removal [10]. We did not see any hardware failures or fractures in this cohort, as have been described elsewhere [10, 11, 24]. An overall complication rate of 14.9% for non-biological (synthetic) fixation of ACJ injury was reported in a systematic review by Borbas et al. in 2019 [24]; again, the BiPOD technique compares favourably with these results.

The results demonstrated significant and persistent restoration of ACJ position radiographically, both in a vertical and horizontal direction on stress radiographs. The well-documented C-C distance showed near perfect reduction of the joint in a vertical plane, compared to the contralateral side, which was maintained up to 2 years following surgery. The AC-DC measurement of vertical ACJ displacement, which has been shown to have greater reliability and resilience to radiographic projectional error than C-C distance [19, 25], also demonstrated statistically and clinically significant restoration of ACJ position at 2 years. Loss of vertical reduction, with increasing C-C distance has been seen to occur with the majority of other stabilisation techniques [10, 11, 24], showing the BiPOD technique in a favourable light against the existing techniques for restoration of vertical stability.

The GC-PC, which provides an objective measure of horizontal displacement of the ACJ, demonstrated excellent restoration of joint position on Alexander lateral stress radiographs at 2 years post-surgery. This additional contribution to horizontal stability from the BiPOD technique is a feature that has not been demonstrated with other techniques; some of the most modern and advanced methods have documented persistence of horizontal stability following surgery, while the majority ignore the issue completely when reporting outcomes [11, 12, 24]. This novel development from the BiPOD technique adds a significant component to the potential biomechanical advantages of this method over the majority of those previously described.

As with all case series, there are inherent limitations to such a study in terms of potential selection bias, lack of a comparator group and a clear outcome measure by which to judge the relative efficacy of the intervention against a standard treatment. In addition, our cohort is relatively small, although comparable to similar research introducing other ACJ stabilisation techniques. We suffered minimal, but material missing data due to the challenges of collecting so many scores and radiographs across several timepoints; our data presentation has been transparent in this respect and statistical analysis altered to account for this limitation. Further research is required to evaluate this method directly against other surgical techniques, monitor results in a larger cohort and to evaluate long-term outcomes.

## Conclusion

In this cohort, the arthroscopically assisted BiPOD technique for ACJ stabilisation demonstrates admirable intermediate term results with excellent clinical outcomes scores and a favourable complication rate compared to existing techniques. The BiPOD technique provides a comprehensive surgical option for the stabilisation of acute ACJ injuries restoring both vertical and horizontal stability.

## Data Availability

All data were available to all members of the research team for review and the senior author (MZ) holds responsibility for the integrity of the data.

## References

[1] Mazzocca AD, Arciero RA, Bicos J (2007). Evaluation and treatment of acromioclavicular joint injuries. Am J Sports Med.

[2] Rockwood CA, Green DP (1984). Fractures in adults.

[3] Keener JD (2014). Acromioclavicular joint anatomy and biomechanics. Oper Techn Sport Med.

[4] Beitzel K, Mazzocca AD, Bak K (1989). ISAKOS upper extremity committee consensus statement on the need for diversification of the Rockwood classification for acromioclavicular joint injuries. Arthroscopy.

[5] Bannister G, Wallace W, Stableforth P, Hutson M (1989). The management of acute acromioclavicular dislocation. A randomised prospective controlled trial. J Bone Jt Surg Br.

[6] Gollwitzer M (1993). Surgical management of complete acromioclavicular joint dislocation (Tossy III) with PDS cord cerclage. Aktuel Traumatol.

[7] Ferris BD, Bhamra M, Paton DF (1989). Coracoid process transfer for acromioclavicular dislocations. Clin Orthop Relat R.

[8] Weaver JK, Dunn HK (1972). Treatment of acromioclavicular injuries, especially complete acromioclavicular separation. J Bone Jt Surg.

[9] Mazzocca AD, Santangelo SA, Johnson ST (2006). A biomechanical evaluation of an anatomical coracoclavicular ligament reconstruction. Am J Sports Med.

[10] Gstettner C, Tauber M, Hitzl W, Resch H (2008). Rockwood type III acromioclavicular dislocation: surgical versus conservative treatment. J Shoulder Elb Surg.

[11] Salzmann GM, Walz L, Buchmann S (2010). Arthroscopically assisted 2-bundle anatomical reduction of acute acromioclavicular joint separations. Am J Sports Med.

[12] Scheibel M, Dröschel S, Gerhardt C, Kraus N (2011). Arthroscopically assisted stabilization of acute high-grade acromioclavicular joint separations. Am J Sports Med.

[13] Hann C, Kraus N, Minkus M (2017). Combined arthroscopically assisted coraco- and acromioclavicular stabilization of acute high-grade acromioclavicular joint separations. Knee Surg Sports Traumatol Arthrosc Off J Esska.

[14] Kumar V, Garg S, Elzein L (2014). Modified weaver-dunn procedure versus the use of a synthetic ligament for acromioclavicular joint reconstruction. J Orthop Surg-hong K.

[15] DeBeer J, Schaer M, Latendresse K (2016). BiPOD arthroscopic acromioclavicular repair restores bidirectional stability. Orthopedics.

[16] Schär MO, Jenni S, Fessel G (2019). Biomechanical comparison of two biplanar and one monoplanar reconstruction techniques of the acromioclavicular joint. Arch Orthop Traum Su.

[17] Zanca P (1971). Shoulder pain: involvement of the acromioclavicular joint. (Analysis of 1000 cases). Am J Roentgenol Radium Ther Nucl Medicine.

[18] Alexander OM (1949). Dislocation of the acromioclavicular joint. Radiography.

[19] Zumstein MA, Schiessl P, Ambuehl B (2017). New quantitative radiographic parameters for vertical and horizontal instability in acromioclavicular joint dislocations. Knee Surg Sports Traumatol Arthrosc.

[20] Fialka C, Oberleitner G, Stampfl P (2005). Modification of the Constant-Murley shoulder score—introduction of the individual relative constant score. Inj.

[21] Fuchs B, Jost B, Gerber C (2000). Posterior-inferior capsular shift for the treatment of recurrent, voluntary posterior subluxation of the shoulder*. J Bone Jt Surg-Am.

[22] Taft TN, Wilson FC, Oglesby JW (1987). Dislocation of the acromioclavicular joint. An end-result study. J Bone Jt Surg.

[23] Welch BL (1947). The generalization of `student’s’ problem when several different population variances are involved. Biometrika.

[24] Borbas P, Churchill J, Ek ET (2019). Surgical management of chronic high-grade acromioclavicular joint dislocations: a systematic review. J Shoulder Elb Surg.

[25] Karargyris O, Murphy RJ, Arenas A (2020). Improved identification of unstable acromioclavicular joint injuries in a clinical population using the acromial center line to dorsal clavicle radiographic measurement. J Shoulder Elb Surg.

